# A Systematic Review Comparing Nonoperative Management to Appendectomy for Uncomplicated Appendicitis in Children

**DOI:** 10.7759/cureus.18901

**Published:** 2021-10-19

**Authors:** Emmanuel Mudika Mosuka, Kalanchige N Thilakarathne, Naushad M Mansuri, Neelam K Mann, Shariqa Rizwan, Afrah E Mohamed, Ahmed E Elshafey, Akanchha Khadka, Lubna Mohammed

**Affiliations:** 1 Medicine, Faculty of Health Sciences, University of Buea, Buea, CMR; 2 Research, California Institute of Behavioral Neurosciences and Psychology, Fairfield, USA; 3 Medical Documentation, Boston Children's Hospital, Boston, USA; 4 Medicine, Nepal Medical College, Kathmandu, NPL

**Keywords:** nonperforated, conservative, pediatric population, children, uncomplicated apendicitis, appendectomy, nonsurgical, nonoperative management

## Abstract

More than a century after its introduction, appendectomy has remained the gold standard treatment for acute appendicitis. In adults with acute uncomplicated appendicitis, nonoperative management (NOM) has been shown to be a viable treatment option. To date, there has been relatively limited data on the nonoperative management of acute appendicitis in the pediatric population. The primary objective of this study was to systematically review the available literature in the pediatric population and compare the efficacy and recurrence between initial nonoperative treatment strategy and appendectomy in children with uncomplicated appendicitis. In July 2021, we conducted systematic searches of the PubMed and Google Scholar databases. We only included full-text comparative original studies published within the last decade, and we excluded articles that solely examined NOM without comparing it to appendectomy. Two writers worked independently on the data collection and analysis. It was found that NOM had a high initial success rate and a low rate of recurrent appendicitis. After months of follow-up, the vast majority of patients with uncomplicated acute appendicitis who received initial nonoperative treatment did not require surgical intervention. Furthermore, the rate of complication was comparable in both treatment groups, and NOM did not appear to be associated with an increased risk of complications. The most significant drawback stemmed from the fact that the included articles in this study had a wide range of study designs and inclusion criteria. According to current evidence, NOM is feasible and cost-effective. Antibiotic therapy can be given safely in a small subset of individuals with uncomplicated appendicitis. To optimize outcomes, physicians should evaluate the clinical presentation and the patient's desire when selecting those to be managed nonoperatively. Again, more research, preferably large randomized trials, is required to compare the long-term clinical efficacy of NOM with appendicectomy. Finally, additional research is required to establish the characteristics of patients who are the best candidates for nonoperative treatment.

## Introduction and background

Rationale

In both adult and pediatric populations, acute appendicitis is one of the most common reasons for emergency abdominal surgery [1]. The lifetime risk of appendicitis is believed to be 7-8%, with the majority of cases occurring in adolescents [2]. Approximately 300,000 appendectomies are performed in the United States of America every year, with 70,000 of those being performed on children, at an average cost of $9000 [3,4]

Appendectomy has long been considered the standard of care for appendicitis management due to its curative success [5]. The belief was that if acute appendicitis is not treated immediately, it will eventually result in perforation. Today, this idea is being challenged, with many researchers advocating for the natural resolution of appendicitis without the need for surgical intervention [6]. Even though open and laparoscopic appendectomies are both safe and simple procedures, operative management is nevertheless associated with a number of problems [7]. Complications from surgery and the risk of anesthesia occur in up to 10% of children who have appendectomy [8]. Furthermore, even in nations with the finest diagnostic techniques, such as the United States of America and Canada, the frequency of negative appendectomy is as high as 4.3-6.3% [9].

There has been an increased interest in the conservative management of appendicitis over the last 20 years [10]. The main benefit of nonoperative treatment is that it avoids the difficulties of surgery and the risk of anesthesia. Additionally, the successful use of antibiotics in treating intra-abdominal infections such as diverticulitis has aroused renewed interest in the nonoperative management of appendicitis [11]. Several systematic reviews and prospective trials have recently explored and demonstrated the efficacy, safety, and cost-effectiveness of antibiotic treatment of adult appendicitis [12-15]. Large randomized controlled trials (RCTs), such as the Appendicitis Acuta (APPAC) trial, have also demonstrated that surgery can be avoided in as many as 72.7% of individuals with uncomplicated appendicitis following one year of follow-up [5]. To date, there has been relatively limited data on the nonoperative management of acute appendicitis in the pediatric population. Despite a paucity of long-term data, the few published meta-analyses and systematic reviews on the conservative treatment of appendicitis in children have revealed encouraging findings. Overall, these trials found that antibiotic therapy in children had a high initial success rate ranging from 62% to 92%, with no significant increase in the rate of complications for those who subsequently underwent interval appendectomy [16-20]. The main goal of this study was to systematically review the available literature in the pediatric population and compare the efficacy, recurrence, cost, and complication between initial conservative treatment strategy and operative treatment strategy in children with uncomplicated appendicitis. 

## Review

Methods

This systematic review was carried out following the Preferred Reporting Items for Systematic Reviews and Meta-Analyses (PRISMA) statement [21].

Eligibility Criteria

We included only full-text studies comparing nonoperative versus surgical therapy of uncomplicated appendicitis in children younger than 19 years. Comparative retrospective or prospective studies and randomized control trials were included if they were published within the recent decade (2011-2021). Nonoriginal articles (systematic review, meta-analysis) and studies focusing on adults were eliminated. Furthermore, studies that simply looked at nonoperative management (NOM) without comparing it to appendectomy were excluded. Additionally, we excluded studies that involved individuals with complicated appendicitis such as perforation, abscess, peritonitis, etc.

Information Sources

We did a comprehensive search of the literature in the PubMed and Google Scholar databases for relevant research published in the last 10 years (2011-2021) to determine which articles were suitable.

Search Strategy

We used the Boolean operators OR and AND in conjunction with the following search terms: uncomplicated, nonperforated, appendicitis, nonoperative, nonsurgical, conservative, operative, surgical, appendectomy, pediatric population, children, and our search were limited to the articles published within the last 10 years.

Study Selection

Two reviewers (ME and TK) did an initial screening, with any discrepancies resolved by our supervisor (ML). After initial screening, full-text publications that matched our inclusion criteria were added to the database.

Data Items and Outcomes

The primary outcome was the initial success rate of nonoperative management compared to appendectomy. We defined the initial success rate in the NOM group as the resolution of acute appendicitis leading to hospital discharge without surgical intervention. While treatment success in the operative treatment group was defined as a successful appendectomy judged as perfect and did not require reoperation. A secondary outcome measure was the recurrence rate, defined as the proportion of patients who required appendectomy or readmission after initial discharge due to recurrent abdominal pain, or the percentage of patients who developed recurrent appendicitis after completing the initial antibiotic course. We also compared the length of the initial hospital stay, the cost of both treatment options, and the postoperative complications reported in the studies.

Results

Study Selection

**Figure 1:**
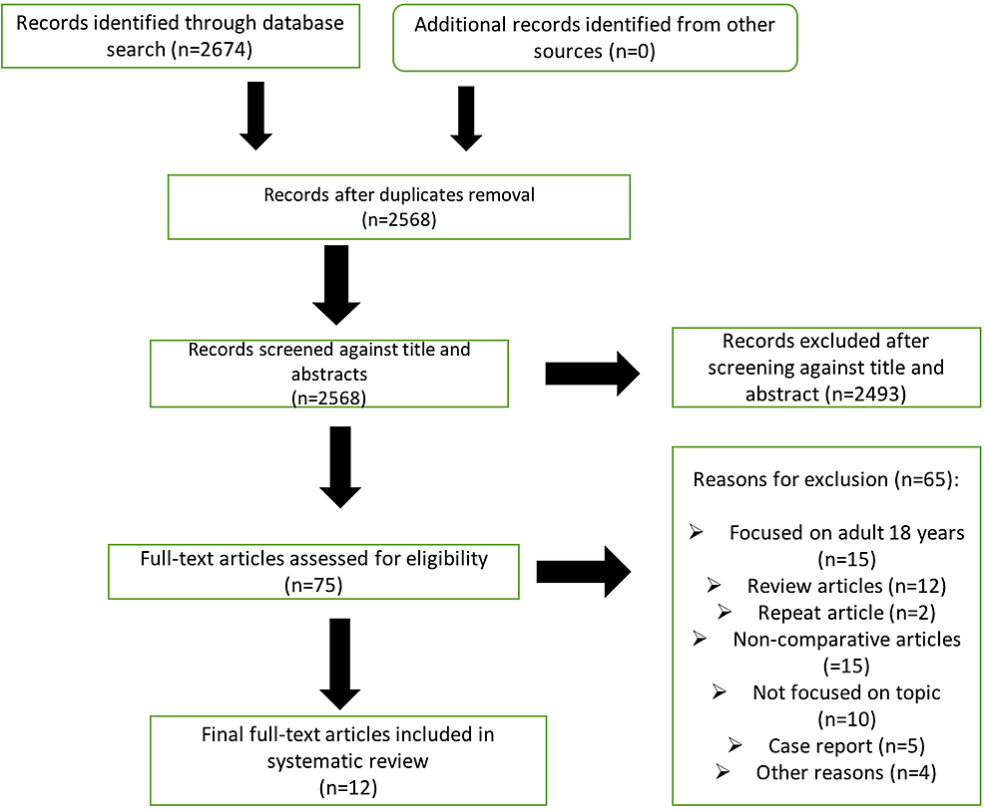
PRISMA flowchart describing the data collection and study selection processes. PRISMA: Preferred Reporting Items for Systematic reviews and Meta-Analyses

Our online process to identify relevant articles for this review is illustrated in the PRISMA chart in Figure 1 below. After a thorough search, we identified and screened 2568 articles. We reviewed 75 full-text articles and excluded 65, leaving 12 final papers in the systematic review.

Study Characteristics

Table 1 summarizes the characteristics of the 12 articles included in this systematic review. Each of these papers examined at least one outcome variable in which nonoperative therapy was compared to appendectomy. This systematic review had more than one randomized control trial, making it the first of its sort in children. Previous research comprised only one RCT, conducted as a pilot study to guide future studies on this topic [22]. In all, 6673 individuals were divided into two groups, the nonoperative treatment group and the appendectomy group. The two RCTs used a computer-based randomization method, whereas the remaining articles reported allocation based on patient or parent preference. Appendicitis was diagnosed in all 12 articles utilizing a combination of clinical, laboratory, and imaging findings. The inclusion/exclusion criteria and the length of the follow-up varied between studies, with the median follow-up ranging from 6.5 months to 52 months. As the primary treatment, all patients in the NOM group received intravenous broad-spectrum antibiotics for 24-48 hours. After that, patients were shifted to oral regimens for the remaining seven to 10 days of therapy. Patients in the appendectomy group got preoperative antibiotics, which were continued for at least 24 hours following surgery in certain studies.

**Table 1:** General features of the included articles. Cipro: ciprofloxacin; metro: metronidazole; NOM: nonoperative management; RCT: randomized controlled trials; PIP: piperacillin-tazobactam

Study	Age group	Study design	NOM(N)	Surgery	Intervention in the NOM group	Follow-up period (months)
Armstrong et al., 2014 [23]	0-18 years	Retrospective	12	12	Cipro and metro	Median - 6.5
Koike et al., 2014 [24]	1-15 years	Retrospective	130	114	Cefoperazone	30.6
Tanaka et al., 2015 [25]	6-15 years	Prospective	78	86	Cefmetazole and ampicillin	Median - 54
Svensson et al., 2015 [22]	5-15 years	RCT	24	26	Meropenem and metro	Minimum - 12
Hartwich et al., 2016 [26]	5-18 years	Prospective	24	50	PIP	Mean - 14
Mahida et al., 2016 [27]	7-17 years	Prospective	5	9	PIP or cipro and metro	12
Minneci et al., 2016 [28]	7-17 years	Prospective	37	65	PIP or cipro and metro	21
Maini et al., 2017 [29]	0-16 years	Prospective	42	32	Ceftriaxone and metro	Minimum - 12
Bachur et al., 2017 [30]	0-19 years	Retrospective	4190	61522	PIP or cipro and metro	12
Mudri et al., 2017 [31]	6-17 years	Retrospective	26	26	Ceftriaxone and metro	36
Lee et al., 2018 [32]	3-17 years	Prospective	51	32	Ceftriaxone and metro	Median - 13
Sajjad et al., 2021 [33]	5-15 years	RCT	90	90	Meropenem and metro	12

Primary Outcome

Initial success rate of treatment: We defined initial treatment success in the antibiotic group as the resolution of acute appendicitis leading to hospital discharge without the need for surgical intervention. As indicated in Table 2 below, we considered 11 articles in our analysis of the initial treatment success rate. NOM had an initial success rate of 90.4%; 469 out of 519 patients with uncomplicated appendicitis were successfully treated initially without the need for surgery. We excluded the multicenter study done by Bachur et al. for determining the initial success rate since they only reported the total success of NOM over a one-year follow-up period with no data on the initial success rate [30].

**Table 2:** Initial success rate of nonoperative management versus appendectomy. NOM: nonoperative management

Study	Initial success rate of NOM	Initial success rate of appendectomy	Recurrence of appendicitis in NOM group
Sajjad et al., 2021 [33]	85/90 (94.4%)	100%	10 (11.8%)
Maini et al., 2017 [29]	36/42 (85%)	100%	6 (16.7%)
Hartwich et al., 2016 [26]	21/24 (87.5%)	100%	2 (9.5%)
Mudri et al., 2017 [31]	23/26 (88.4%)	100%	9 (39%)
Svensson et al., 2015 [22]	22/24 (92%)	100%	6 (27%)
Tanaka et al., 2015 [25]	77/78 (98.7%)	100%	22 (28.6%)
Koike et al., 2014 [24]	125/130 (96.2%)	100%	24 (19.2%)
Armstrong et al., 2014 [23]	10/12 (83.3%)	100%	5 (50%)
Minneci et al., 2016 [28]	33/37 (94.6%)	98.5%	5 (15.1%)
Lee et al., 2017 [32]	35/51 (68.6%)	100%	9 (25.7%)
Mahida et al., 2016 [27]	2/5 (40%)	100%	Abandoned
Total	469/519 (90.4%)	21%	541/542 (99.8%)

Initial failure rate associated with appendicolith: Five studies demonstrated that appendicolith was related to a significant likelihood of initial treatment failure [22,25,27,30,33]. Sajjad et al. noted that all five patients (100%) who failed initial treatment had appendicolith [33]. Mahida et al. reported three cases of treatment failure with appendicolith on imaging [27]. So, due to the high initial failure rate, they halted the study out of worry for the patients' safety. Lee et al. observed a similar finding; seven out of 14 (50%) of NOM patients with appendicolith failed initial conservative treatment, compared to 24% without it [32]. In a study by Svensson et al., 60% of NOM patients with appendicoliths eventually had surgery [22]. According to Tanaka et al., appendicolith was indicative of total NOM failure. They discovered that 47% (9 of 19) of those with appendicoliths in the NOM group experienced treatment failure, compared to 24% of those who did not [25]. In addition, Sajjad et al. discovered a higher initial failure rate in individuals with elevated C-Reactive protein and leukocyte counts [33].

Recurrence of Appendicitis

We defined the recurrence rate as a percentage of patients who experienced recurrent appendicitis after completing the initial antibiotic course. Table 2 above shows the recurrence of appendicitis in the NOM group; 21% of the 469 patients who had a successful initial antibiotic treatment experienced recurrence appendicitis during the follow-up period. The multicenter study by Bachur et al. noted a high recurrence of 46% during one year of follow-up [30]. Still, we omitted this article in calculating the overall recurrence since they relied mainly on administrative data. Koike et al. noticed that appendicolith was associated with a high rate of recurrence [24]. They reported that out of 24 patients with recurrence, five had appendicolith. Also, three of nine patients with recurrence in the study by Mudri et al. had fecalith [31]. However, Sajjad et al. noticed no association; they noted that none of their ten patients with recurrence had appendicolith [33].

Length of Hospital Stay

Eight studies provided comparative data on the length of hospital stay (LOS) [22-25,28-32]. Three of these studies revealed that the NOM group had a longer LOS than the appendectomy group [22,28,31]. Svensson et al., for example, reported a median LOS of 34.5 hours and 51.5 hours for the nonoperative and surgical groups, respectively [22]. However, the other five studies found that there was no statistically significant difference in total LOS between the two groups.

Cost of Treatment

Six studies provided data on cost for nonoperative and surgical treatment options [22,24,26,28,31,32]. Three of the six studies reported significantly lower appendicitis-related total costs for those treated conservatively [24,26,28]. For example, Hartwich et al. reported $1359 savings for every patient treated nonoperatively compared to the surgical group [26]. Though the other three articles noted a lower initial inpatient cost for nonoperative management, the total cost was similar for both groups.

Complication of Treatment

In total, we included 10 articles in the comparative analysis of the rate of complication in both groups. As indicated in Table 3, the percentage of children who experienced one or more complications ranged from 0 to 18.1% for NOM and 0 to 28% for the surgical group. The multicenter study, which used administrative data from 45 tertiary pediatric hospitals across the United States of America, revealed that those treated conservatively had more advanced imaging, hospitalization, and emergency department visits during the one-year follow-up period [30]. With regards to severe complications as a result of initial conservative therapy, two perforations were seen. Also, Mahida et al. noted that none of the three cases which failed conservative treatment had complicated appendicitis at the time of surgery [27].

**Table 3:** Rate of complication of NOM versus appendectomy. NOM: nonoperative management; SSI: surgical site infection; SBO: small bowel obstruction; ED: emergency department

Study	Complications from nonoperative management	Complications from appendectomy
Maini et al., 2017 [29]	7.4%: 2 cases of SSI and 1 postoperative adhesive bowel obstruction	28.12%: 5 cases of wound infection, 1 abscess, 1 chest infection and, 2 bowel obstruction
Hartwich et al., 2014 [26]	None	None
Mudri et al., 2017 [31]	None	15%: 2 cases of intra-abdominal abscess, nausea, vomiting, and 1 case of clostridium difficile colitis
Svensson et al., 2015 [22]	1 perforation	None
Tanaka et al., 2015 [25]	1 perforation	2.3%: 2 cases of ileus
Armstrong et al., 2014 [23]	8.3% (1 of 2): 1 deep SSI	16.7% (2/12): deep and superficial SSI
Minneci et al., 2016 [28]	2.7% (1/37)	7.7% (5/65): readmission and reoperation cases
Lee et al., 2017 [32]	18% (9/51): 1 intraoperative drain, 1 adhesive SBO	19% (6/32): 2 cases of postoperative ileus, 1 SSI infection, 1 intrabdominal abscess, and 1 case of constipation
Bachur et al., 2017 [30]	NOM patients were more likely to have subsequent imaging, hospitalizations, and ED visits than patients in the appendectomy group	Less

Discussion

Over the years, surgery has been the standard of care for acute appendicitis in children and adults. Despite extensive research in adult patients, little is known about the feasibility and safety of initial nonoperative therapy in children with uncomplicated appendicitis when compared to appendectomy. This review found that there is a scarcity of relevant evidence in children, which is consistent with previous findings. Our study is the first to incorporate two RCTs in its analysis; the pilot trial with 50 participants and the article with 180 patients done by Sajjad et al. [33]. The objective of this study was to systematically review available literature in the pediatric population and compare the efficacy and recurrence between initial nonoperative treatment strategy and appendectomy in children with uncomplicated appendicitis. Our analysis showed a high initial success rate (90.4%) of nonoperative management. About 72% of those treated nonoperatively avoided appendectomy after a follow-up period of six months to 52 months. Also, this review suggests that nonoperative management was more cost-effective compared to appendectomy. The length of hospital stay and rate of complication was similar in both groups.

A prior systematic review and meta-analysis by Huang et al. compared NOM with appendectomy and indicated that nonoperative therapy was related to a high initial success rate with a frequency of 90.5%, which is nearly identical to this review. Like our study, they included only comparative articles in their systematic review [17]. In addition, Maita et al. revealed in another meta-analysis that NOM had a high initial success rate, with an efficacy of 92% [18]. They used data from 21 studies in their analysis; 13 of them were comparative studies, while the remaining eight were studies that exclusively provided outcomes of NOM. Another systematic review by Kessler et al. found that appendectomy had a high overall success rate (98%), with 74% of their NOM patients avoiding surgery after follow-up [16]. Finally, according to the findings of a systematic study undertaken by Gorter et al., approximately 62-81 % of children who were treated nonoperatively did not require appendectomy after follow-up [19].

Association of Appendicolith and Initial Failure Rate

Nonoperative management proponents differ on whether or not patients with appendicoliths should be included in the NOM group or if they should undergo surgery as soon as possible instead. According to the findings of this study, the presence of an appendicolith is associated with a greater chance of NOM failure and recurrence. Similar findings were demonstrated in the systematic review and meta-analysis by Huang et al., who reported a significant initial nonoperative treatment failure rate for individuals with appendicolith [17]. A possible explanation for this is the fact that an appendicolith raises the chance of luminal occlusion, which in turn increases the risk of perforation.

The Recurrence Rate of Acute Appendicitis

According to this study, the recurrence rate of acute appendicitis is low ( 21% ) following initial successful nonoperative therapy. Sajjad et al. found an 11.8% recurrence rate (10/85), with histology confirming acute appendicitis in all cases [33]. However, Svensson and colleagues reported that only one out of seven patients who later underwent interval appendectomy exhibited acute appendicitis on histology [22]. Despite a 92% initial success rate, Maita and colleagues' meta-analysis demonstrated a low recurrence rate, with just 16% of patients in the NOM group requiring interval appendectomy [18]. Similarly, Huang and colleagues observed a 16.1% recurrence rate one-year follow-up.

Comparing Cost of NOM with Appendectomy

This review reveals that patients treated nonoperatively have much reduced appendicitis-related initial costs. This could be explained by the high success rate (90.4%) of initial nonoperative management, which reduces additional follow-up costs. According to Harwich et al., nonoperative treatment saved $1359 on average per patient compared to surgery [26]. Minneci et al. also discovered that the total health cost of antibiotic treatment was much cheaper than that of appendectomy, with a median cost of $4219 against $5029 [28]. However, three out of six studies revealed that the total cost of both therapies was comparable [22,31,32].

Length of Hospital Stay

The extended length of hospital stay is another concerning issue among proponents of nonoperative treatment of appendicitis. This review suggests that NOM is associated with an increased length of hospital stay. Svensson et al. observed a median LOS of 34.5 hours for the nonoperative group and 51.5 hours for the surgical group [22]. Similarly, Minneci et al. reported a median LOS of 20 hours and 37 hours for appendectomy and NOM, respectively [28]. The fact that some studies required patients enrolled in NOM to be discharged only when they were able to accept oral feeds could account for the longer length of stay (LOS) in the NOM group.

Delayed Appendectomy and Rate of Complication

Delaying an appendectomy, according to NOM critics, increases the risk of complications including perforation, which can be life-threatening. However, a systematic review by Xu et al. provided evidence that delayed appendectomy does not significantly increase perforation nor complication rates [20]. This viewpoint is supported by the evidence from this review, as only two incidences of perforation were recorded from the NOM group. Besides, these were all early treatment failures that might not be related to treatment type but the condition at initial diagnosis. It could have been caused by a mistaken initial assignment of patients to the NOM group who should have been in the appendectomy group. To reduce complication rates from initial nonoperative management, careful clinical evaluation combined with patient preference should be used when assigning patients to either group.

Limitations

The most significant limitation of this systematic review was that the 12 included studies had a diverse set of study designs and inclusion criteria. For example, the decision to include or exclude patients with appendicoliths had a varying impact on the outcomes of the study. The interventions employed in the articles also differed; the antibiotic regimen utilized in the NOM group varied amongst included articles. Lastly, the rate of recurrence could also have been influenced by the different follow-up durations used in the included articles. Additionally, the rate of recurrence could have been influenced by the studies' varying follow-up periods. The longer the follow-up period, the more cases of recurrent appendicitis are likely to occur. Finally, in most articles, parents' choice was used to assign patients to the NOM group. As important as the parent's choice is, there is a tendency to promote selection bias, as a parent with a severely ill child will likely choose appendectomy rather than NOM.

## Conclusions

According to current evidence, NOM is feasible and cost-effective. Antibiotic therapy can be given safely in a small subset of individuals with uncomplicated appendicitis. The rate of complication was comparable in both treatment groups, and NOM did not appear to be associated with an increased risk of complications. To optimize outcomes, physicians should evaluate the clinical presentation and the patient's desire when selecting those to be managed nonoperatively. Again, more research, preferably large randomized trials, is needed to compare the long-term clinical efficacy of NOM to appendicectomy. Finally, additional research is required to establish the characteristics of patients who are the best candidates for nonoperative treatment.
